# Next-generation sequencing: insights to advance clinical investigations of the microbiome

**DOI:** 10.1172/JCI154944

**Published:** 2022-04-01

**Authors:** Caroline R. Wensel, Jennifer L. Pluznick, Steven L. Salzberg, Cynthia L. Sears

**Affiliations:** 1 Department of Medicine and; 2 Department of Physiology, Johns Hopkins University School of Medicine, Baltimore, Maryland, USA.; 3 Department of Biomedical Engineering,; 4 Department of Computer Science, and; 5 Department of Biostatistics, Johns Hopkins University, Baltimore, Maryland, USA.; 6 Department of Oncology, Johns Hopkins University School of Medicine, Baltimore, Maryland, USA.

## Abstract

Next-generation sequencing (NGS) technology has advanced our understanding of the human microbiome by allowing for the discovery and characterization of unculturable microbes with prediction of their function. Key NGS methods include 16S rRNA gene sequencing, shotgun metagenomic sequencing, and RNA sequencing. The choice of which NGS methodology to pursue for a given purpose is often unclear for clinicians and researchers. In this Review, we describe the fundamentals of NGS, with a focus on 16S rRNA and shotgun metagenomic sequencing. We also discuss pros and cons of each methodology as well as important concepts in data variability, study design, and clinical metadata collection. We further present examples of how NGS studies of the human microbiome have advanced our understanding of human disease pathophysiology across diverse clinical contexts, including the development of diagnostics and therapeutics. Finally, we share insights as to how NGS might further be integrated into and advance microbiome research and clinical care in the coming years.

## Introduction

The number of microbial cells that reside on and in us rivals the number of our own cells (1). In health, we, the host, and microbes live in symbiosis. However, many illnesses are defined by or associated with microbial dysbiosis. These include both communicable diseases such as tuberculosis (2) and syphilis (3), and non-communicable diseases like inflammatory bowel disease (4), diabetes (5), obesity (6), and cancer (7). We have known since the time of Koch and the discovery of *Mycobacterium tuberculosis* that our microbial inhabitants affect our health status. However, how we characterize these organisms has drastically changed (8–10). Koch’s postulates laid a framework for assessing microbial causes of disease through culturing methods. Indeed, Koch’s postulates are still relevant, more than 130 years after they were first published; however, we now also recognize the importance and vast diversity of unculturable microbes (11). Advances in technology like next-generation sequencing (NGS) have led to an explosion in the discovery and characterization of microbes, because NGS methods do not rely on traditional culture techniques and can thus detect the unculturable microbes (Table 1). In fact, complementing of traditional culture methods with NGS has already been implemented in many clinical microbiology laboratories because of its potential to address severe, insidious infections (12). Advantages of NGS include its ability to identify more unique species than traditional culture methods, and the capacity to perform parallel sequencing of multiple samples, which, with the earlier, low-throughput Sanger sequencing technology, was not technically feasible.

The purpose of this Review is to discuss crucial features relating to NGS in translational research and clinical care. We first discuss the fundamentals of NGS and compare common methodologies as well as sources of data variability and important study design considerations. Then, we present select examples of how NGS has altered our collective understanding of disease pathogenesis. Finally, we offer insights as to how NGS might further be integrated into and advance clinical care in the coming years with the aim of helping researchers and clinicians consider the impact of NGS on disease diagnostics and therapeutics.

## Fundamental considerations in the use of ngs

*What are the fundamentals of NGS?* An initial question in studying the gut microbiota is which microbes are present in a given sample. Subsequent inquiries, addressable by NGS analyses, include determining the relative abundance and predictive functional profiles of the microbes present, as well as understanding intraspecies and population heterogeneity (13). NGS methods address these questions by directly sequencing microbial DNA or RNA, for example, in fecal, blood, and/or tissue samples. With the improving affordability of NGS, the two primary NGS methodologies now in use are amplicon sequencing and shotgun metagenomic sequencing; however, RNA sequencing is also a valid and, in some ways, superior method for microbial characterization, as it allows for determination of the transcriptome, representing a further step to define microbiota function (14, 15).

**Figure 1:**
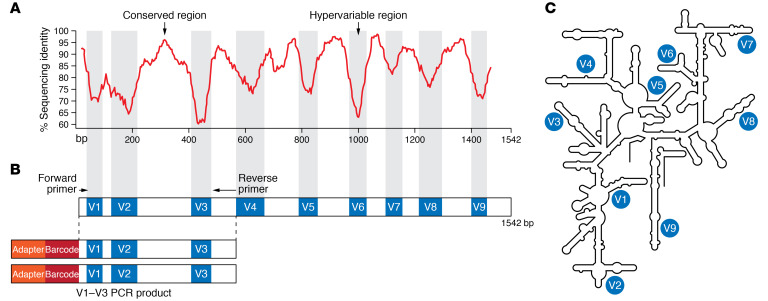
Bacterial 16S rRNA gene. (**A**) Percentage sequence identity of conserved and hypervariable regions of the bacterial 16S rRNA gene. Adapted with permission from the *Journal of Microbiological Methods* (17) and Ilona Lehtinen (137). (**B**) Illustration of conserved and hypervariable regions corresponding to **A** and PCR amplification of the V1–V3 region of the bacterial 16S rRNA gene. Adapted with permission from Humana Press (148). (**C**) Schematic of 16S rRNA gene structure with hypervariable regions (V1–V9) labeled.

One of the most common NGS methods for bacterial identification and characterization is amplicon sequencing. Amplicon sequencing involves first amplifying a region of the DNA via PCR, and then sequencing the resultant product. The target for PCR amplification is, most commonly, the bacterial 16S ribosomal RNA (rRNA) gene (Figure 1). For this reason, amplicon sequencing is also referred to as 16S rRNA sequencing or analysis. The use of the 16S rRNA gene to characterize uncultured microbes was first described by Lane et al. in 1985 (16). The 16S rRNA gene is an ideal target because it is highly conserved and ubiquitous among bacteria (without it, bacteria would be unable to translate mRNA into proteins and thus be nonfunctional) and it also contains nine hypervariable regions (V1–V9) that differ between bacterial species and genera (Figure 1). Thus, PCR primers can be designed such that forward and reverse primers bind to conserved regions but amplify an intervening variable region. Typically only a subset of the variable regions are targeted for sequencing in a given study (e.g., V1–V3, V4–V5) to limit the amount and, thus, time and cost of sequencing. However, it is important to note that no one region adequately differentiates all bacteria (17), and sequencing of select hypervariable regions can yield differing data interpretation (17–19). For example, amplification of certain hypervariable regions may bias results, leading to under- or overrepresentation of taxa (18), but may also be advantageous for distinguishing between certain species within a genus (17). Recently, NGS sequencing of the full 16S rRNA gene has emerged and, using increasingly sophisticated analytical methods, may provide both species and strain resolution in microbiota communities (20).

After PCR amplification of the selected hypervariable regions, the resulting amplicons are sequenced, followed by data “cleaning.” Data cleaning involves multiple steps, such as adapter and primer sequence trimming, removal of low-quality bases and sequences from reads, and removal of sequences matching a control library such as the PhiX Control (Illumina), chimeric sequences, and human contaminant reads, as well as chloroplast and mitochondrial contaminants. Subsequent analyses lead to organization of the sequence data into, most often, operational taxonomic units (OTUs). OTUs are distance-based clusters of sequences, initially constructed without a reference database (21). An OTU sequence identity greater than 97% (or with up to 3% dissimilarity) is typically estimated to define a species, while OTUs with sequence similarities of 95% and 80% are used to define genus and phylum, respectively (21). Taxonomic identification is then inferred by computational alignment to reference 16S rRNA sequence databases such as the Ribosomal Database Project (RDP) (22), SILVA (23), or Greengenes (24). OTUs and identified taxa are then used for downstream analysis. An alternative, less frequently used non-distance-based analytical approach for amplicon sequencing relies on exact nucleotide matching to yield amplicon sequence variants (ASVs). ASV taxon assignments are dependent on the quality of reference databases (25). Additionally, ASVs have the potential to split single genomes into multiple clusters, because most bacterial cells possess more than one rRNA gene copy and these, not infrequently, differ in nucleotide sequence (26). While each method (OTUs versus ASVs) has proponents (26, 27), importantly, both are computational approaches to estimate taxonomy. For unculturable microbes, NGS data alone produce “candidate species,” whereas firmer classification of cultured bacterial species is possible using both phenotypic and genome sequence data (28).

**Figure 2:**
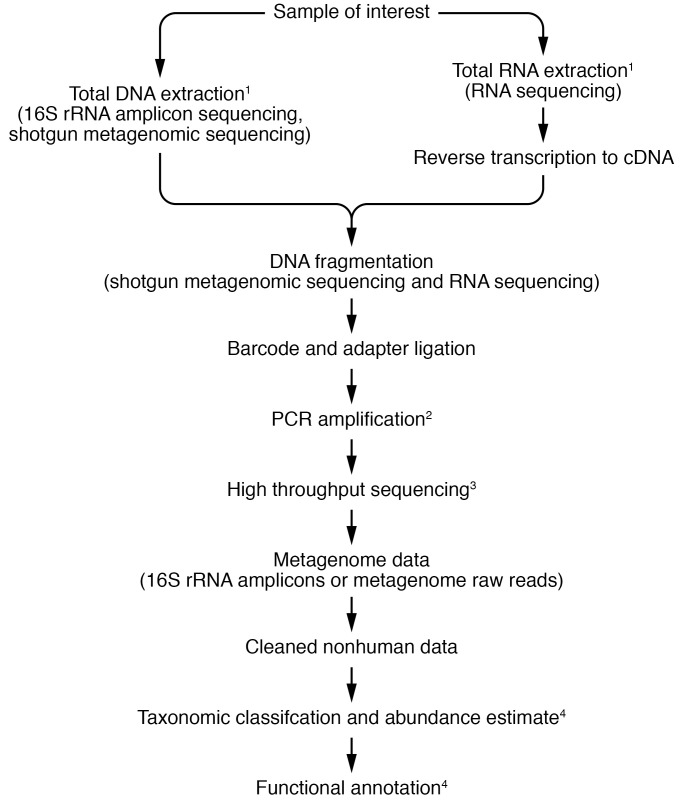
NGS implementation. Overview of key steps in 16S rRNA gene sequencing, shotgun metagenomic sequencing, and RNA sequencing processes. ^1^Host DNA or RNA depletion can be performed (optional steps). ^2^PCR amplification is used to amplify bacterial 16S rRNA gene variable regions (16S rRNA amplicon sequencing) or random cDNA fragments resulting from RNA reverse transcription for RNA sequencing. DNA-based shotgun metagenomic sequencing is optimally done without use of PCR amplification to avoid introduction of PCR-associated experimental bias. However, in samples with low DNA quantities, PCR amplification of the DNA library is sometimes used. ^3^Commonly Illumina-based sequencing chemistry (33). 4The taxonomic and functional analyses of NGS data are complex and make use, most often, of software available in the public domain.

In contrast to amplicon sequencing, shotgun metagenomic sequencing and RNA sequencing analyze all the DNA or RNA in a given sample, respectively. For shotgun metagenomic sequencing, after extraction, the DNA is randomly fragmented, and barcodes and adapters are ligated to the ends of each segment to facilitate sample identification and DNA sequencing. The resultant reads are cleaned and subsequently aligned to a reference database to identify taxa and functional potential. The primary reference databases are usually Reference Sequence (RefSeq; ref. 29) and GenBank (30). These are large databases containing all publicly available genomes. Smaller pathogen-focused databases such as Pathosystems Resource Integration Center (PATRIC; ref. 31) and the Eukaryotic Pathogen Database (EuPathDB; ref. 32) are also used. The RNA sequencing workflow is similar to that for shotgun metagenomic sequencing; however, after fragmentation, the RNA segments are reverse transcribed, using PCR, into complementary DNA (cDNA), which is then processed using the DNA sequencing pipeline. Figure 2 provides an overview of NGS processes. Because of their diverse methodologies, 16S rRNA amplicon, shotgun metagenomic, and RNA sequencing each have advantages and drawbacks. These are discussed below. Choosing one method over the others requires comparison and consideration of study goals. Several recent reviews and books have provided guides to microbiome analysis (21, 33).

### Direct comparisons between NGS methods.

Although comparisons of 16S rRNA sequencing and shotgun metagenomics exist for a variety of samples, including those from humans (14, 15, 34–40), laboratory model organisms (13, 39), plants (39, 41), soil (42), and water (43), overall, direct method comparisons for human samples are limited. Comparisons with RNA sequencing and across all three sequencing modalities are even more limited (14). Here, we address common considerations in choosing an NGS method (Table 2). Additionally, we review studies within the past five years that have directly compared NGS methods in humans (Table 3).

16S rRNA, shotgun metagenomic, and RNA sequencing can all be used to determine what bacteria are present in a microbiome; however, the latter two also detect members of other domains such as fungi and parasites, as well as viruses. Only RNA sequencing examines RNA viruses. With respect to taxonomic resolution, an overarching finding of the studies that have compared these methods is that phylum designations are comparable (39); however, 16S rRNA sequencing tends to offer less resolution and sensitivity for detecting changes at the species level and cannot detect strain-level changes (13, 34, 43). For example, Jovel and colleagues conducted parallel 16S rRNA and shotgun metagenomic sequencing on mock bacterial populations with defined consortia and found that the 16S rRNA method and software pipelines (Quantitative Insights Into Microbial Ecology [QIIME], refs. 44, 45; and mothur, ref. 46) effectively resolved sequences to the genus level, but shotgun metagenomic sequencing resulted in improved genus- and species-level classification (36). This finding has been replicated in other human studies (Table 3 and refs. 14, 15, 35–40). Interestingly, Drewes et al. compared 16S rRNA analysis pipelines and found that the Resphera Insight high-resolution taxonomic assignment tool (Resphera Biosciences; refs. 47–49) better characterized species-level differences using human colon cancer samples compared with other 16S rRNA sequencing pipelines (50). To our knowledge, no studies, as yet, have directly compared the Resphera Insight (47–49) pipeline species classification with that of shotgun metagenomic sequencing. Very recently, the Kraken pipeline for shotgun metagenomic analysis was expanded to enable 16S rRNA analysis, and results show that it is more accurate and up to 300 times faster than QIIME (51). However, QIIME includes a wealth of other helpful tools, making it more a stand-alone “complete” package. For users sophisticated enough to mix-and-match packages, Kraken could replace the core QIIME step of 16S read assignment.

A functional profile cannot be directly obtained from 16S rRNA sequencing, because the method only characterizes sequences from one essential gene. Methods like PICRUSt (Phylogenetic Investigations of Communities by Reconstruction of Unobserved States; ref. 52) or PICRUSt2 (53) and Tax4Fun (54) or Tax4Fun2 (55) aim to predict functional profiles of bacteria based on 16S rRNA data. However, the success of these methods, when compared with functional potentials obtained via shotgun metagenomics, varies with the 16S gene primers used for amplification (35, 36). Conversely, shotgun metagenomics and RNA sequencing consider all the microbial DNA and RNA; thus it is possible to more comprehensively predict the functional potential. Importantly, a distinct difference between shotgun metagenomics and RNA sequencing is that shotgun metagenomics provides a random selection of all genes encoded by the microbes (predictive functional potential) whereas RNA sequencing identifies which genes are actively being transcribed (active functional profile).

Other considerations in pursuing an NGS method and analyses include host contamination, false positives, bias, and post-sequencing computational requirements. There is less risk of host contamination in 16S rRNA sequencing compared with other NGS methods because the gene being amplified and sequenced (i.e., the 16S rRNA gene) is specific to bacteria. With 16S rRNA sequencing, there is also a lower risk of false positives due to extensive reference databases and computational error correction tools; however, the risk of false positives increases with decreasing sample biomass (33). Conversely, there is a higher risk of bias with 16S rRNA sequencing because of primer-dependent PCR amplification bias and differences between the variable regions, as discussed above (17–19). Importantly, one must also consider the computational expertise and analysis required after sequencing. Currently, 16S rRNA sequencing bioinformatics analysis is less of an undertaking than either shotgun metagenomics or RNA sequencing, as there are fewer data (i.e., sequencing output from one gene versus all genes) as well as several publicly available and user-friendly platforms, like QIIME (44, 45) and mothur (46). This makes 16S rRNA sequencing more accessible to researchers with beginner- and intermediate-level bioinformatics experience (33). For projects directed at detection of specific taxa, pilot data using mock microbial communities can guide experimental choices (e.g., primer sets and/or estimation of read numbers or sequencing depth [see below] needed for taxon identification).

Finally, cost must be considered for any project and is arguably one of the most important factors in what type of NGS to initially perform. The differences in cost between the methods relate to the amount and depth of sequencing. Sequencing depth refers to the number of times a certain nucleotide base is represented in the sequencing reads for a given sample (56). Typically, shotgun metagenomics and RNA sequencing analyses require much more sequence data than 16S rRNA sequencing, resulting in their higher costs. However, a recent study by Laudadio and colleagues suggests that shotgun metagenomics, at lower sequencing depths, is comparable in price to 16S rRNA sequencing and still identifies more species (38). Notably, this study did not consider other inherent NGS costs, including computational burden and data storage.

In summary, the use of the 16S rRNA gene as a phylogenetic marker is efficient and cost effective (52); however, it is subject to biases that other microbiome characterization methods are not (i.e., choice of hypervariable regions and primer-dependent PCR amplification) and can thus result in significant variance in the determined microbial composition of a sample. Additionally, 16S rRNA sequencing is commonly limited to taxonomic classification at the genus level or above (36), as horizontal transfer of the 16S rRNA locus and the existence of multiple bacterial species and strains that are more than 97% similar can prevent more nuanced classification (35, 43). Finally, 16S rRNA analysis provides limited predicted functional information (14, 52). Conversely, shotgun metagenomics and RNA sequencing are more expensive than 16S rRNA sequencing but offer far broader taxonomic coverage (i.e., species- and strain-level resolution), more accurate functional profiling, and the possibility of detecting previously unknown species and strains of microbes (36). Although shotgun metagenomic and/or RNA sequencing undoubtedly provides more information, determining which approach is appropriate depends on the question(s) being asked. For instance, if you want to identify the dominant bacteria in a sample, 16S rRNA sequencing is likely the better method owing to the lower cost and bioinformatics burden (42). We present comparisons herein not to suggest that one sequencing method or protocol is best for all projects but rather to assist readers in selecting the best protocol for their projects.

### Technical and individual laboratory issues: sources of variability.

There are multiple parameters to consider regarding sample collection and processing, because variabilities in any of these steps can alter NGS data. First, the investigator must choose the type of sample for NGS sequencing. Although fecal samples and body fluids are easier to collect and permit serial sampling, intraluminal fecal samples or tissue samples may provide representative regional colon or site-specific microbiome characterization. Storage conditions can further impact NGS results, and thus this information should be reported. The gold standard is immediate freezing of samples and storage at –80°C (57); however, samples can also be preserved chemically using solutions such as DNA/RNA Shield (Zymo Research) (58).

The first step in sample processing is DNA or RNA extraction, and this step is responsible for the majority, but not all, of experimental variability in microbiome analysis according to the MicroBiome Quality Control project (59). Numerous commercially available kits exist for DNA extraction, including from Covaris, Qiagen, Zymo Research, and others. Typically samples are homogenized, but protocols vary substantially from laboratory to laboratory (59). Although there is not yet a globally accepted gold-standard protocol for DNA or RNA extraction, it is critical that all samples be processed in the same manner. Furthermore, it is strongly recommended that negative controls be processed to better assess the comparability of different NGS runs, normalize across separate NGS runs to limit batch effects, identify kit-specific contaminants, and determine whether the detection of low-abundance microbes in a sample are of biologic interest or, more likely, represent contaminants. Examples of controls include (a) storage buffer (e.g., DNA/RNA Shield); (b) DNA extraction kit components; and (c) a community standard containing known species at known quantities (e.g., Zymo Microbial Community Standard [D6300] and Zymo Microbial Community Standard II Log Distribution [D6310]).

For 16S rRNA sequencing, the PCR amplification step is also a source of variability. As discussed, there are nine hypervariable regions in the 16S rRNA gene, and available primer sets typically amplify only a subset of these regions. Thus, the performance characteristics of the primer set chosen will influence the number of the analyzable reads (60) as well as the results of the analysis (61). For example, one study reports that the V4 primer set yields significantly more *Bacteroides* and lower Firmicutes reads than other primer sets tested; this is particularly notable given that the Bacteroidetes/Firmicutes ratio is a commonly reported metric (60).

The results of sequencing itself also vary with different equipment, and thus, ideally, all samples are sequenced using the same sequencing platform (e.g., Illumina MiSeq, NovaSeq). Finally, a wide variety of bioinformatics pipelines are available, for both 16S rRNA and shotgun metagenomics data, and the choice of computational and statistical methods can have a critical effect on outcomes and conclusions (36, 59, 62), including the risk of reporting false associations and of missing true ones. While a full review of computational methods and their relative strengths and weaknesses is beyond the scope of our discussion, Liu et al. (33) provided a recent review covering dozens of methods.

Overall, variability in any of the steps of NGS sequencing (e.g., sample type, sample storage, DNA extraction, PCR amplification, sequencing technology, read length, and/or bioinformatics analysis) can lead to data variability. There is generally not a “right” answer as to the best method or approach. The most important principle is that all samples be treated the same to facilitate meaningful comparisons between samples in the same study. However, as discussed in the next section, great care must be taken in comparing results between different studies, as these variables may differ.

### Challenges of rigor, reproducibility, and reporting.

Microbiome science is complex, cutting across many scientific fields, including microbiology, epidemiology, biology, computational science, genomics, and biostatistics. This complexity and the rapid evolution of approaches within the field have led to the reporting of disparate findings between studies investigating seemingly similar patient populations. Thus, increasing attention is now directed to developing well-curated and validated databases that are critical for accurate analyses, and providing guidance for the consistent conduct and reporting of study design, methods, and results of microbiome research. In 2018, Schloss provided a thoughtful and pragmatic essay for translational researchers to consider the threats to rigor, reproducibility, and generalizability within microbiome research (63). Others have called for a centralized robust curated data repository for microbiome data adherent to FAIR (findable, accessible, interoperable, and reproducible) principles (64). Consistent with this need, the FDA has established an evolving quality-controlled and highly curated public microbial reference database (FDA-ARGOS) for microbiome research (65), although this database is still relatively small. Most recently, the STORMS (Strengthening the Organization and Reporting of Microbiome Studies) 17-point Microbiome Reporting Checklist was proposed as a guide for researchers, reviewers, and readers for the presentation, assessment, and understanding of microbiome research across studies (66, 67). Although STORMS was developed through a strong iterative process, it is based on the analysis of only one paper and has been minimally used to date (66). Nonetheless, previous reporting guidelines — e.g., CONSORT (Consolidated Standards of Reporting Trials) — improved the quality of clinical trial reporting (66), and such results support calls for more structured microbiome research reporting. Improvement of microbiome science communication and of the ability to cross-compare studies is essential for human microbiome studies to yield progress in applying microbiome science to patient care.

### Essential considerations in collection of clinical metadata.

Beyond the complexities of designing the laboratory, computational, and statistical approaches to NGS-driven human studies, the investigator must also consider what and how much clinical metadata to collect. Age, sex, and geography are fundamental as each impacts microbiome composition and likely function (68, 69). However, given the interindividual variability in the microbiome (70) and disease-associated data (discussed below), more nuanced considerations of individual exposures, both current and over time, may be needed. These include environmental exposures associated with migration (71), diet and food additives (72), and antibiotic and non-antibiotic medications (73, 74). While genetic impacts on the human microbiome have been downplayed in recent literature (75), this is likely short-sighted, as we do not yet understand how microbial communities function, and data suggest that select members of the microbiome serve as functional drivers that intersect with host genes to regulate clinical outcomes (76–78). This broad field of human exposures that impact health and disease is termed the “exposome” and, while impossible to fully capture in most studies, deserves careful thought in study design, data accrual, and interpretation (79).

## Relevance of ngs to clinical and translational research

Although extremely useful in investigating disease mechanisms that may inform human translational research, herein, we will not consider the enticing but likely overinterpreted rodent microbiome studies (80). Instead, given the breadth of available data, we focus on a few illustrative examples of human microbiome analyses to indicate the robust impact that NGS-derived microbiome data can have on our thinking about human diseases; such results implicate the potential for human microbiome science to impact clinical care (81).

### Nutrition and metabolism.

In the very active area of investigation concerning nutrition and metabolism (82), we provide a few seminal observations that may help guide considerations in NGS microbiome and implementation research. Only key highlights from each paper are presented, and the reader is referred to the individual publications for further details. In 2011, Wu and colleagues provided human data strongly linking diet and gut microbiome composition (83). This feeding study identified that a diet change (low fat/high fiber versus high fat/low fiber) led to detectable gut microbiome changes within 24 hours, without perturbation of overall compositional microbiome structure. These data provide insight into rapid diet-dependent microbiome shifts, but suggest that long-term diet is key to overall gut microbiome structure and likely function. O’Keefe and colleagues demonstrated that a mere 2-week switch in diet, from a US-based Western diet (high fat/low fiber) to a rural African diet (low fat/high fiber) or vice versa, led to remarkable reciprocal changes in mucosal inflammation and proliferation as well as metabolic health indicators in African American and rural South African populations (84). In a very detailed study of individual diet and health impact, Zeevi and colleagues identified, unexpectedly, the wide variability in human diet metabolic processing and physiologic impact. Namely, postprandial glycemic response to identical meals and combination foods like pizza varies dramatically between individuals, but can be predicted using a machine learning algorithm that integrates microbiome NGS data and other inputs (85). For example, pizza may not significantly alter the metabolism of one person but may induce hyperglycemia in another. Lastly, the Gordon laboratory, based on at least a decade of investigations integrating rodent-based experimental and human studies, provided the first microbiota-directed complementary food prototype, termed MDCF-2. This proof-of-principle, prospective, randomized study was conducted in a population of children with moderate acute malnutrition in Bangladesh. It yielded strong evidence that a diet composed of locally sourced foods could improve weight-for-length *z* scores in 3 months but also identified that the *z* scores declined quickly after MDCF-2 supplementation withdrawal (86). It remains to be determined whether this diet, locally sourced from Bangladeshi foods, will yield similar results in other global locales and/or whether the MDCF development process can be streamlined to yield products able to promote global nutritional health equity.

### The skin in health and disease.

The skin microbiota is essential for protection against invading pathogens. One remarkable aspect of the skin microbiome is its regional diversity whereby the local (e.g., ear versus navel) microbiome varies, possibly because of differential environmental exposures (70). The accessibility of the skin and ease of sampling have fostered exemplary longitudinal studies (needed in other areas of microbiome science) of conditions like atopic dermatitis. This work provides insight into the skin microbiome and disease-associated bacterial strain fluctuations, although disease mechanisms require further study (87, 88).

### Early life exposures.

Microbiome analyses of birth cohorts and early-life exposures underpin our fundamental understanding of development (89), exposure impacts (e.g., cesarian versus vaginal delivery [ref. 90], antibiotics [ref. 91]), and disease onset (e.g., childhood asthma, atopy [ref. 92]). This work highlights that the early-life fecal compositional assembly and metabolome associate with the emergence of childhood atopy and asthma years later, in part because of immune development dysregulation (92, 93). It also underpins the importance of microbiome analyses for the prediction of disease development in additional, large, longitudinal birth cohort studies. One example is a study of 100,000 mother-baby pairs in the Greater Bay Area in China, led by the Faculty of Medicine at the Chinese University of Hong Kong (64).

### Defining outbreak transmission, source, and pathogenicity.

NGS studies, both whole-genome and metagenomic sequencing, along with detailed epidemiologic analyses have been instrumental in tracking and identifying the source of multi-drug-resistant pathogens, persistent even over extended time periods in a hospital (94, 95). Pathogen identification enabled interventions to eliminate the infection source and understand hospital spread. Furthermore, NGS studies of outbreak human *Burkholderia* strains, isolated from individuals with cystic fibrosis, led to the identification of bacterial genes promoting this bacterium’s human host adaptation and virulence (96). Identifications such as these offer insights for new therapeutic targets.

### Impact of NGS on disease diagnosis.

A clinical benefit of microbiome NGS may be to predict disease risk, akin to the established use of human genome NGS to identify disease risk. Microbiome NGS to predict disease risk is not yet validated for any disease, but progress is occurring. For example, as described above, longitudinal studies in children have begun to link microbes to risk for onset of asthma and atopic conditions (92, 93). Another example is the use of the colon microbiome (i.e., colon mucosal or fecal samples) to predict colorectal cancer (CRC) risk. To date, although metagenomic analyses detect microbial communities reflective of CRC, detection of communities reflective of precancerous lesions (e.g., colonic polyps) is limited (97, 98). Similarly, blood-based transcriptomes best detect advanced-stage cancers (99). This suggests that NGS methods need further development to detect early-stage disease when intervention may enhance patient prognosis (100).

The clinical microbiology laboratory is beginning to use microbial NGS methods for disease diagnosis, particularly to identify potential infectious etiologies of chronic illnesses. Use of NGS has emerged to define undiagnosed CNS infections (12, 101, 102), respiratory pathogens (103), and other difficult-to-diagnose or undiagnosed infectious diseases. Metagenomic sequencing is attractive for detecting suspected, but undiagnosed, infections because nucleic acid analyses can, theoretically, detect bacteria, viruses, fungi, and parasites. This extensive detection potential could limit the numerous tests required to assess a broad array of putative pathogens in patients without diagnoses. Hurdles include differentiating colonization from infection, limiting contaminants, developing efficient, clinical sample–specific methods, achieving analytical standardization, and continually working to improve data security to protect patient privacy. Cost is another hurdle, as NGS method validation can be very expensive (12).

Research is needed to define how use of microbiome NGS can advance patient care, solve the source of outbreaks (e.g., *Klebsiella* [ref. 94] and *Sphingomonas* [ref. 95]), and identify and characterize emerging pathogens (e.g., SARS-CoV-2 [ref. 104]). The most difficult challenge for clinicians and translational scientists is the interpretation of NGS data for clinical application (12, 105, 106).

## Development of therapeutics from NGS and the microbiome

To date, there are no FDA-approved therapeutics based on NGS or derived from the human microbiota or microbiome. Nonetheless, this is a rich area of research, and we highlight some important and ongoing work in the next sections.

### Whole community transfer: fecal microbiota transplantation.

Fecal microbiota transplantation (FMT) is an ancient therapy (107), having been employed as early as the 4th century BCE in China, and has been proposed as the method most likely to succeed in manipulating pathophysiologically complex diseases (108). FMT is used primarily for the treatment of *Clostridioides difficile* disease but is also being explored, with variable clinical outcomes, as a therapeutic for other gastrointestinal (109, 110) and non-gastrointestinal diseases (111, 112). Key limitations of FMT, as currently used, are its inherent lack of quality control and imprecision, combined with our weak understanding of the microbes and mechanisms by which FMT may confer benefit. Furthermore, enthusiasm for the use of FMT is now more restrained with the emergence of SARS-CoV-2 (live virus is present in feces; refs. 113, 114), safety concerns (115, 116), deaths (117), FDA warnings (118), and more stringent screening requirements (119). Recent analysis, with improved delineation of variables impacting FMT, indicates that its outcomes, even with *C*. *difficile* disease, may not be as robust as previously suggested by the case report literature (116, 120). However, promising microbiome community–based quality-controlled FMT products (e.g., SER-109, in ECOSPOR clinical trials; and RBX2660, in PUNCH clinical trials; ref. 121), based, at least in part, on NGS, are being studied in prospective, randomized clinical trials (SER-109, ref. 122; RBX2660, refs. 123, 124; >400 studies at ClinicalTrials.gov, accessed November 27, 2021). Most recently, a phase III, double-blind, placebo-controlled trial of SER-109, an oral microbiome therapeutic composed of human stool–derived live Firmicutes bacterial spores, reported efficacy superior to that of placebo in lowering rates of *C*. *difficile* recurrence in all age groups studied (recurrence, SER-109 vs. placebo, 12% vs. 40%; relative risk, 0.32; 95% confidence interval, 0.18–0.58, *P <* 0.001). The safety profile was similar to placebo (125). FMT and microbial replacement products require more study to understand the mechanisms, microbes, and durability by which these therapeutics alter gut microbiome function and drive clinical outcomes (126).

### Additions to the host microbiome: prebiotics and probiotics.

Both untargeted and targeted approaches to modulate the function of the microbiome are being studied, each of which utilizes and/or requires NGS to assess impact. The first and most prevalent untargeted example over time has been the ingestion of over-the-counter prebiotics and probiotics. Prebiotics are substrates (e.g., fiber) that are consumed by gut microbes, whereas probiotics are live organisms ingested to confer health benefits. Because most prebiotics and probiotics are not subject to regulatory oversight, the products can be highly variable (127). Recent data, for example, have demonstrated a lack of benefit of the most commonly used probiotic globally, *Lactobacillus rhamnosus* (LGG or R0011), studied with or without *L*. *helveticus* R0052, in childhood diarrhea (128, 129). Furthermore, the colonization by and effect of probiotic strains appear to vary significantly between individuals (82). Nonetheless, another recent study, using fecal microbiome analyses of individuals in rural Thailand, identified that *Bacillus*, a spore-forming bacterium, was associated with reduced human *Staphylococcus aureus* colonization, a cause of systemic antibiotic-resistant infections. This outcome was ascribed to *Bacillus* production of lipopeptides (fengycins) that inhibit *S*. *aureus* quorum-sensing mechanisms (130). Thus, development of rational precision probiotics based on NGS and microbiome research is likely feasible and is another key microbiome research opportunity.

### Preclinical targeted NGS-linked tactics to modulate the microbiome.

Multiple approaches, under development, may allow for precision manipulation of the gut microbiome and have potential to impact local and/or systemic disease processes. These include (a) inhibition of gut bacterial enzymes to modify metabolic capabilities; (b) selective bacteriophage-mediated depletion of disease-inducing or undesirable bacterial strains; (c) gut colonization with engineered strains that deliver a therapeutic payload; and (d) direct genetic modification of the in situ microbiome (131). Excitingly, human proof-of-principle studies already exist for some of these approaches. For example, bacteriophages have been successfully used to treat systemic antimicrobial-resistant infections (132, 133), and an engineered, oral *E*. *coli* Nissle strain promoted arginine synthesis from ammonia in healthy volunteers (134) and is being developed as a potential treatment for hyperammonia conditions (e.g., hepatic encephalopathy).

### The microbiome as a source of new drugs.

Many antibiotics, including penicillin, are natural products or their derivatives. As an extension of this prior success, high-dimensional, multicomponent screening strategies have recently been used to identify antimicrobials with novel mechanisms of action from uncultured soil or marine microbiome members (135, 136). Whether these microbiome derivatives will be successful in humans remains to be tested.

## Conclusions

Microbiome science is moving toward microbiome precision medicine, but we still lack sufficient clinical data, either cross-sectional or longitudinal, to apply the science to human health or disease with confidence. Investigative needs include integrative human-centric microbiome studies, broader and more consistent integration of the exposome, a better understanding of the putative unique contributions of different analytical approaches, and cross-validation of data sets between studies of disease processes and populations. Ultimately, NGS results will be complemented with non-NGS methods such as metabolomics and proteomics to understand microbial functions in health and disease. Critically, data must be interpreted with consideration of clinical plausibility. Furthermore, and in parallel, investigators should be explicit about gaps in knowledge or new directions for additional clinical studies.

Application of NGS data and microbiome investigations in clinical medicine is in its infancy, and thus contains both promise and uncertainty. The current paucity of carefully designed prospective and longitudinal human studies highlights a rich opportunity for clinical translational scientists. By leveraging the cross-disciplinary nature and complexities of microbiome science, we can advance our understanding of disease development, progression, diagnosis, and therapy, ultimately benefiting the health of patients.

## Figures and Tables

**Table 1:**
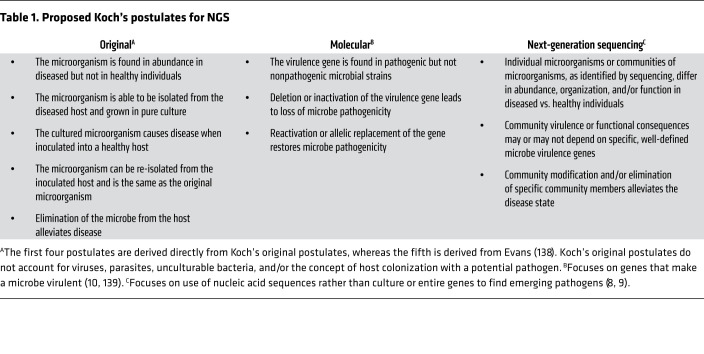
Proposed Koch’s postulates for NGS

**Table 2:**
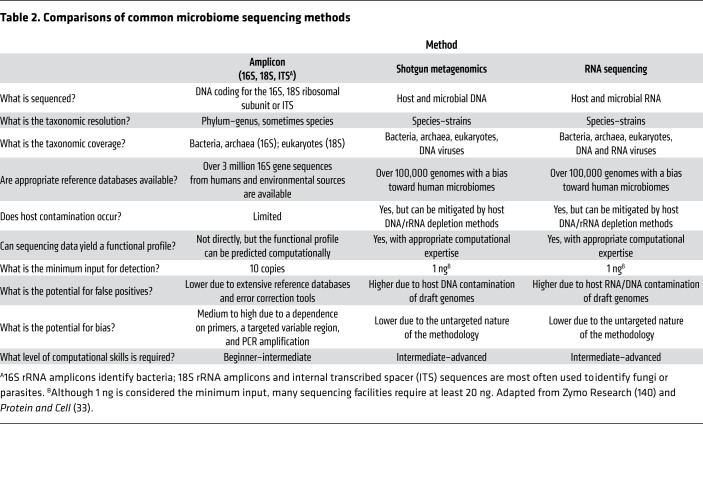
Comparisons of common microbiome sequencing methods

**Table 3:**
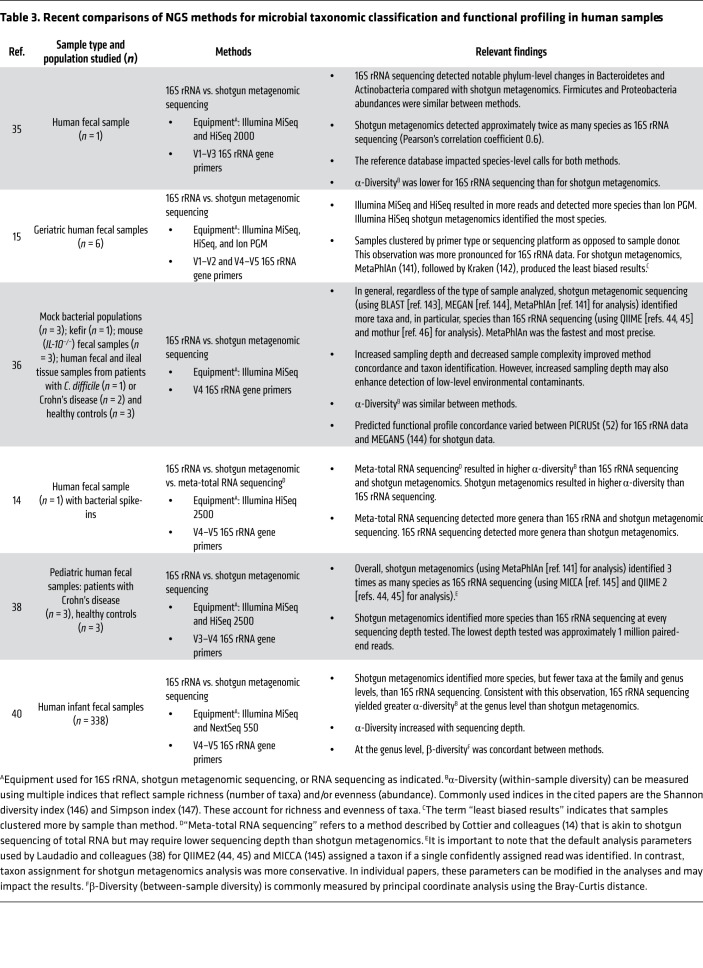
Recent comparisons of NGS methods for microbial taxonomic classification and functional profiling in human samples

## Version 1 04/01/2022

Electronic publication
